# Skipping breakfast and physical fitness among school-aged adolescents

**DOI:** 10.6061/clinics/2020/e1599

**Published:** 2020-07-15

**Authors:** Jingcen Hu, Zhifei Li, Sixuan Li, Hui Li, Sijia Wang, Shuyu Wang, Lei Xu, Delun Yang, Tiecheng Ruan, Hang Li, Shuo Han, Qinghai Gong, Liyuan Han

**Affiliations:** I Department of Epidemiology, Zhejiang Provincial Key Laboratory of Pathophysiology, School of Medicine, Ningbo University, Ningbo, China; II Department of Pediatric, the Affiliated Hospital of Medical School, Ningbo University, Department of Pediatricthe Affiliated Hospital of Medical SchoolNingbo UniversityChinaChina; III Ningbo Municipal Center for Disease Control and Prevention, Ningbo, China; IV Medical Insurance Department, Ningbo Medical Center Lihuili Hospital, Ningbo, China

**Keywords:** Breakfast Skipping, Physical Fitness, Adolescents

## Abstract

**OBJECTIVES:**

This study explored the relationship between skipping breakfast and physical fitness in a group of school-aged adolescents in China.

**METHODS:**

This cross-sectional study from the Chinese National Surveillance on Students’ Constitution and Health (CNSSCH) survey in Ningbo, China, used a standardized questionnaire to assess the frequency of breakfast consumption. Physical fitness was measured through standing long jump, 50-m sprint, 1,000 (or 800)-m run, and vital capacity tests. Multiple linear regression analysis was used to investigate the relationship between the frequency of breakfast consumption and physical fitness.

**RESULTS:**

Our study included a total of 1,849 school-aged adolescents (aged 15.53±1.80 years). Among boys, non-breakfast-skippers had good scores for 50-m sprints, 1,000-m run, and vital capacity tests when compared with breakfast skippers (all *p*<0.05). Among girls, non-breakfast-skippers had a good scores for the standing long jump test compared with breakfast skippers (*p*=0.003). The multiple linear regression model showed that not skipping breakfast was positively associated with vital capacity (β=-173.78, *p*=0.004) and inversely associated with 50-m sprint (β=-0.12, *p*=0.018) and 1,000-m run times (β=-8.08, *p*=0.001) in boys.

**CONCLUSION:**

The results of this cross-sectional study revealed that skipping breakfast might be associated with lower physical fitness in Chinese adolescents aged 13-18 years, especially boys. Breakfast consumption should be promoted among Chinese school-aged boys.

## INTRODUCTION

Adolescence is a critical period for the development of physical health. Regular breakfast consumption is important for ensuring healthy growth and development in adolescents. Mounting evidence has shown positive associations between skipping breakfast and obesity, diabetes, cognitive ability, and academic performance (1-4). An international study found that irregular breakfast consumption rates ranged from 27.4% to 62.2% in adolescents (5).

Few studies have investigated the relationship between breakfast consumption frequency and physical fitness in adolescents (6-11). In a group of 860 British adolescents, eating breakfast was associated with higher moderate and vigorous physical activity on weekends (6). In another group of 877 British adolescents, less frequent breakfast consumption was associated with lower physical activity levels during the morning in girls (7). However, the relationship between breakfast consumption and physical fitness remains controversial. Adolescents who tended to skip breakfast were more likely to have lower levels of physical activity (6,7). Another study conducted by Baldinger et al. Switzerland revealed that people who ate breakfast almost every day had higher physical test scores (shuttle, 20 meter dash, and standing long jump) than those of people who occasionally or never ate breakfast (8). In addition, a study performed in primary school children revealed a positive correlation between the frequency of breakfast consumption and muscle strength (9). However, other studies found no correlations between breakfast consumption and physical fitness (10,11).

The Chinese National Survey on Students’ Constitution and Health (CNSSCH), a national cross-sectional study conducted in 2014, has been widely recognized by international experts for the establishment of internationally accepted student evaluation criteria (12). As an important part of the CNSSCH, the survey on students’ physical health is jointly organized and led by the Ministry of Education, the National Health and Family Planning Commission, and other departments. The investigation on students’ physical health has played an important role in analyzing the characteristics of children’s growth and development in China and in understanding the prevalence of and factors influencing common diseases among students (13).

Furthermore, to date, no study has investigated the relationship between breakfast consumption and physical fitness (including run, sit-ups, grip, and jump) in school-aged adolescents in China. Thus, our study aimed to investigate the relationship between breakfast consumption and physical fitness in Chinese school-aged adolescents from the CNSSCH.

## METHODS

### Study population

This cross-sectional study was part of the CNSSCH study (14) performed in 2014 in Ningbo. Detailed information on the CHNS study design has been published elsewhere (15). A cluster sampling method was used to include children and adolescents from four schools in the urban region of Ningbo. A standardized questionnaire was used to collect data on baseline demographic and lifestyle factors from the past year. The present analyses included data from healthy adolescents aged 13-18 years from high schools. Verbal informed consent was obtained from all participants and their parents. The study was approved by the ethics committee of Ningbo Municipal Center for Disease Control and Prevention.

### Breakfast consumption frequency and other lifestyle factors

Breakfast consumption was assessed by the question. “How many days do you eat breakfast per week?” The answer categories were “≤2 days per week,” “3-5 days per week” or “≥6 days per week.” As only a few subjects (1.63%) reported they ate breakfast ≤2 days per week, the first two categories were combined for analysis. Therefore, the participants were classified as “breakfast skippers” (0-5 days per week) or “non-breakfast-skippers” (≥6 days per week). Milk intake or the frequency of eating eggs was assessed with one item: “how many days do you eat eggs/drink milk per week?” According to the established classification (9,16), the answers were divided into three groups: (1) ≤2 days per week, (2) 3-5 days per week, and (3) ≥6 days per week.

Watching TV or using computer and physical activity were assessed with one item: “In general, how long do you watch TV or use the computer or perform physical activity every day?” The response options included “less than 30 min per day,” “30 to 60 min per day,” and “more than 60 min per day.”

### Anthropometric measurements

Well-trained physicians followed standard procedures to conduct anthropometric measurements. Height (cm) and weight (kg) were measured. Weight was measured to the nearest 0.1 kg in light clothing, using a calibrated beam scale. Height was measured to the nearest 0.1 cm without shoes, using portable stadiometer. Both the scales and stadiometers were calibrated before use. Body mass index (BMI) was calculated as weight (kg) divided by height (m) squared (kg/m^2^).

### Measurement of physical fitness

Physical fitness was measured after lunch in school. The tests included standing long jump, 50-m sprint, 1,000 (800)-m run [boys (girls)], grip strength, and vital capacity.

Two strength tests were performed: 1. standing long-jump (cm) (the best record of three jumps); 2. grip strength (kg) (measured four times, each hand measured twice, with the average value of the highest strength for each hand recorded as the grip strength). Two tests were used to measure running capability, including 50-m sprint (seconds), defined as the time required to sprint 50 m; 1,000(800)-m run (seconds): endurance run, defined as the time taken to run 1000 m for boys and 800 m for girls. One test was used to measure the forced vital capacity (FVC) (mL); forced expiratory measurements were performed twice using electronic spirometers, with the participants in a standing position and the maximum value of the two measurements recorded in milliliter. All tests were performed by trained physical education teachers.

### Statistical analysis

Continuous variables with normal distribution were expressed as means ± standard deviation (SD) and were analyzed using t-tests. Chi-square tests were used to analyze categorical variables, and the results were expressed as frequencies (percentages). Tests were used to compare the differences between boys and girls as well as the difference in physical fitness stratified by different groups. Multiple linear regression was used to investigate the associations between breakfast consumption frequency and physical fitness in boys and girls. Two-sided *p*<0.05 was considered significant. All analyses were performed using SPSS Statistics for Windows, version 17.0 (SPSS Inc., Chicago, IL, USA).

## RESULTS

### Baseline characteristics of the study participants

The descriptive characteristics of all study participants are shown in Table 1. Our study enrolled a total of 1,849 school-aged adolescents with a mean age of 15.53±1.80 years, including 959 (51.87%) boys and 890 (48.13%) girls. The proportions of boys and girls who skipped breakfast were 13.2% and 11%, respectively. We observed significant differences between boys and girls in weight, height, BMI, milk intake, and breakfast consumption frequency (all *p*<0.05). Compared with girls, boys had a shorter sleep duration, higher percentages for breakfast consumption frequency <6 days/week (13.2% in boys *vs*. 11% in girls), and lower percentages for milk intake frequency ≤2 days/week (40.7% in boys *vs*. 47.0% in girls).

### Associations between breakfast consumption frequency and physical fitness in different groups

**Figure 1:**
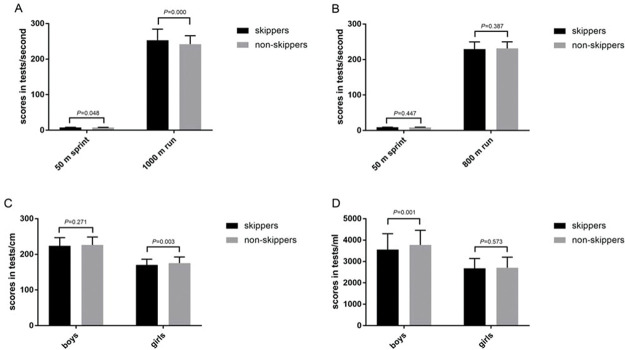
Associations between breakfast consumption frequency and physical fitness in boys and girls (A) Associations between breakfast consumption frequency and running tests in boys. Breakfast skippers (<6 days per week), n=127; Non-breakfast-skippers (≥6 days per week), n=832. (B) Associations between breakfast consumption frequency and running tests in girls. Breakfast skippers (<6 days per week), n=98; Non-breakfast-skippers (≥6 days per week), n=792. (C) Associations between breakfast consumption frequency and standing long jump in boys and girls. (D) Associations between breakfast consumption frequency and vital capacity in boys and girls.

The associations between breakfast consumption frequency and physical fitness stratified by watching TV, computer use, and milk intake in boys and girls are shown in Table 2. Among boys, non-breakfast-skippers had better scores for 50-m sprints, 1,000-m run, and vital capacity tests than did breakfast skippers (all *p*<0.05). Figure 1 illustrates the main results. Boys with milk intake ≥200 mL/day, physical activity ≥30 min/day, TV watching <30 min/day, and computer use <30 min/day had better 1,000-m test scores compared to their counterparts (all *p*<0.05). In addition, girls with milk intake ≥200 mL/day, physical activity≥30 min/day, TV watching <30 min/day, or computer use <30 min/day had better test scores for standing long jump (all *p*<0.05).

### Multiple linear regression analysis of the associations between breakfast consumption frequency and physical fitness in boys and girls

The results of multiple linear regression analyses of the associations between breakfast consumption frequency and physical fitness in boys and girls are shown in Table 3. For boys, skipping breakfast was inversely associated with 50-m sprint and 1,000-m run (β=-0.12, *p*=0.018 and β=-8.08, *p*<0.004, respectively) times and was positively associated with vital capacity (β=173.78, *p*=0.001) after adjusting for age, BMI, milk intake, physical activity time, TV watching, and computer use.

## DISCUSSION

To our knowledge, this is the first study to explore the relationship between breakfast consumption frequency and physical fitness in a group of school-aged adolescents in China. As part of the CNSSCH, this survey was strictly performed according to the standardized procedure. Multiple linear regression was also used to control for confounding factors. Consistent with our findings, other studies also confirmed an increased breakfast consumption frequency and physical fitness (20-m shuttle run, squat jump, and muscle power) in primary school children (9,17).

The results of a previous study suggested, that compared with breakfast skippers, adolescents who regularly ate breakfast had a lower BMI and decreased risks of overweight and obesity (8,18). However, a study by Abalkhail (19) showed that skipping breakfast did not differ by age, sex, or BMI. No differences in BMI were observed between breakfast skippers and non-breakfast-skippers. Few school-aged adolescents were overweight/obese in this study (169/15 in the total sample). The low proportion of adolescents who skipped breakfast (12.2%) might have contributed to the non-significant association between BMI and skipping breakfast.

Our regression analysis indicated that among boys non-breakfast-skippers had higher physical fitness levels (including standing long jump, 50-m sprint, 1,000-m run and vital capacity) than breakfast skippers. However, significant findings were not observed in girls. Previous studies on the relationship between skipping breakfast and physical activity also revealed inconsistent findings regarding sex (7,10,20). One study suggested that boys were generally more physically active than girls and that breakfast consumption may be more important for boys (20). Although physical fitness is more accurate than physical activity, the former can be explained by other factors (17,21). The different health effects of skipping breakfast between boys and girls are probably explained on the basis of biology and physical activity (22).

We also evaluated the relationship between breakfast consumption and vital capacity. Several studies have also examined the association between dietary patterns and ventilator function or cardiorespiratory fitness (23-25). Sandercock et al. found that men who ate breakfast had high levels of cardiopulmonary function, whereas women who ate breakfast had no better cardiorespiratory function than women who skipped breakfast (23). Likewise, we also found that male breakfast skippers were less likely to have a high vital capacity.

The mechanisms linking breakfast consumption and physical fitness have yet to be explored. Studies have shown that adolescents who regularly eat breakfast have higher energy consumption; thus, their BMI remains lower than or similar to that of adolescents who do not eat breakfast (23,26,27). In addition, other studies have confirmed that children who regularly eat breakfast have a higher intensity of physical activity (7,23,28) and lower risks of overweight and obesity (29,30).

This study has some limitations. Owing to the cross-sectional nature of the study design, our data cannot infer causality. There may also be residual confounding factors that were not adjusted for in our study. In addition, breakfast consumption was self-reported and dietary intake was not comprehensively evaluated in this study; thus, the quality of breakfast could not be investigated. Furthermore, it is unclear whether the findings of our study can be extended to other populations, as CNSSCH data collection was carried out in schools. Finally, the CNSSCH survey only included three questions about diet (breakfast consumption, milk intake, and egg consumption), and no nutritional questionnaire was administered.

## CONCLUSION

In summary, skipping breakfast was associated with lower physical fitness, especially in boys. Breakfast promotion should be advocated in boys. Further longitudinal studies are needed to explore the causal relationships.

## AUTHOR CONTRIBUTIONS

Han S, Gong Q and Han, L conceived the project. Li S, Li H and Wang S designed the questionnaire. Xu L, Ruan T and Li H performed the questionnaire. Wang S and Yang D analyzed the data. Hu J and Li Z wrote the manuscript.

## Figures and Tables

**Table 1:** Baseline characteristics in all participants, boys, and girls.

	All participants	Boys	Girls
Participants	1,849	959	890
Age, years (mean±SD)	15.53±1.80	15.57±1.83	15.49±1.78^a^
Weight, kg (mean±SD)	55.35±9.95	59.98±10.08	50.82±7.51^a^ †
Height, cm (mean±SD)	165.48±8.13	170.68±6.64	159.88±5.41^a^ †
BMI, kg/m^2^ (mean±SD)	20.13±2.71	20.38±2.83	19.85±2.55^a^ †
Overweight (obesity)	169 (15)	112 (12)	57 (3)
Milk intake (%)			
≤2 days/week, n=390	43.7	40.7	47.0^b^ †
≥3-5 days/week, n=569	56.3	59.3	53.0
Physical activity time (%)			
<30 min/ day	62.3	62.6	62.0^b^
≥30 min/day	37.7	37.4	38.0
Time spent watching TV (%)			
<30 min/day	74.5	75.7	73.3^b^
≥30 min/day	25.5	24.3	26.7
Time spent using a computer (%)			
<30 min/day	48.8	48.2	49.4^b^
≥30 min/day	51.2	51.8	50.6
Breakfast consumption frequency (%)			
Breakfast skippers (<6 days/w)	12.2	13.2	11.0^b^
Non-breakfast-skippers (≥6 days/w)	87.8	86.8	89.0

BMI, body mass index.

a Differences between continuous variables were analyzed using t-tests.

b Differences between categorical variables were analyzed using chi-square tests.

† *p*<0.05.

**Table 2:** Associations between breakfast consumption frequency, milk intake, physical activity, TV watching, computer time, and physical fitness in boys and girls.

Characteristics	50-m sprint (s) (mean±SD)	Standing long jump (cm) (mean±SD)	1,000-m run (boys) and 800-m (girls) (s) (mean±SD)	Vital capacity (mL) (mean±SD)
Boys, n=959				
Breakfast consumption frequency				
Breakfast skippers (<6 days/w), n=127	7.61±0.64†	223.89±22.93	253.06±31.40†	3,556.25±741.47†
Non-breakfast-skippers (≥6 days/w), n=832	7.51±0.53	226.25±22.44	242.02±23.85	3,773.95±682.02
Milk intake				
≤2 days/week, n=390	7.56±0.60	224.43±22.56	248.50±29.25†	3,707.53±737.334
≥3-5 days/week, n=569	7.49±0.50	226.98±22.43	240.04±21.43	3,773.62±661.32
Physical activity				
<30 min/day, n=600	7.51±0.57	226.73±22.95	245.20±26.41†	3,771.02±692.51
≥30 min/day, n=359	7.53±0.50	224.63±21.71	240.61±22.90	3,701.82±694.54
TV watching				
<30 min/day, n=726	7.50±0.54	226.74±22.08	241.40±23.64†	3,801.26±676.35†
≥30 min/day, n=233	7.58±0.57	223.44±23.65	249.97±28.77	3,570.19±719.08
Computer use				
<30 min/ day, n=462	7.50±0.52	226.75±21.65	239.72±22.13†	3,782.38±705.31
≥30 min/day, n=497	7.54±0.57	225.19±23.27	246.98±27.37	3,710.48±681.65
Girls, n=890				
Breakfast consumption frequency				
Breakfast skippers (<6 days/w), n=98	9.01±0.57	170.27±16.16†	229.66±20.07	2,682.56±456.79
Non-breakfast-skippers (≥6 days/w), n=792	8.96±0.55	175.61±17.08	231.39±18.40	2,710.43±489.95
Milk intake				
≤2 days/week, n=418	9.03±0.58†	172.58±17.03†	234.15±19.90†	2,708.60±498.48
≥3-5 days/week, n=472	8.91±0.52	177.19±16.80	228.58±19.95	2,706.27±475.67
Physical activity				
<30 min/day, n=552	9.01±0.57†	174.16±22.95†	233.82±18.22†	2,700.23±483.67
≥30 min/day, n=338	8.89±0.50	176.42±21.71	226.91±18.42	2,791.00±491.24
TV watching				
<30 min/day, n=652	8.95±0.55†	176.52±17.16†	230.26±18.43†	2,728.55±475.97†
≥30 min/day, n=238	9.03±0.56	170.92±16.10	233.77±18.83	2,649.32±509.87
Computer use				
<30 min/day, n=902	7.50±0.52†	226.75±21.65†	239.72±22.13†	3,782.38±705.31
≥30 min/day, n=947	7.54±0.57	225.19±23.27	246.98±27.37	3,710.48±681.65

SD, standard deviation.

Data were expressed as means and standards deviations and were analyzed using t-tests.

† *p*<0.05.

**Table 3:** Multiple linear regression of the associations between breakfast consumption frequency and physical fitness in boys and girls.

	50-m sprint (s)		Standing long jump (cm)		1,000-m run (boys), 800-m run (girls) (s)		Vital capacity (mL)	
Model†	β (se)	*p*	β (se)	*p*	β (se)	*p*	β (se)	*p*
Boys (n=959)								
Unadjusted	-0.10 (0.05)	0.048	2.36 (2.14)	0.271	-11.04 (2.38)	<0.001	217.70 (65.75)	0.001
Model^a^	-0.15 (0.05)	0.004	4.95 (2.04)	0.016	-9.74 (2.43)	<0.001	210.06 (60.99)	0.001
Model^b^	-0.12 (0.05)	0.018	3.84 (2.05)	0.061	-8.08 (2.42)	0.001	173.78 (60.80)	0.004
Girls (n=890)								
Unadjusted	-0.05 (0.06)	0.447	5.33 (1.82)	0.003	1.72 (1.99)	0.387	27.87 (52.09)	0.593
Model^a^	-0.03 (0.06)	0.595	4.16 (1.79)	0.020	1.26 (1.89)	0.505	8.99 (50.05)	0.857
Model^b^	-0.01 (0.03)	0.828	3.12 (1.79)	0.080	1.95 (1.91)	0.307	1.98 (50.40)	0.969

a Adjusted for body mass index (BMI) and age;

b Same as Model 1+milk intake frequency+physical activity time+TV watching+computer use.

† Reference category: breakfast skippers.
